# The interpretation of polycrystalline coherent inelastic neutron scattering from aluminium

**DOI:** 10.1107/S0021889813023728

**Published:** 2013-10-26

**Authors:** Daniel L. Roach, D. Keith Ross, Julian D. Gale, Jon W. Taylor

**Affiliations:** a Physics and Materials Research Centre, University of Salford, The Crescent, Salford, Greater Manchester M5 4WT, United Kingdom; b Nanochemistry Research Institute, Department of Chemistry, Curtin University, PO Box U1987, Perth, Western Australia WA6845, Australia; c ISIS Facility, STFC Rutherford Appleton Laboratory, Chilton, Didcot, Oxfordshire OX11 0QX, United Kingdom

**Keywords:** polycrystalline coherent inelastic neutron scattering

## Abstract

A new approach to the interpretation and analysis of coherent inelastic neutron scattering from polycrystals (poly-CINS) is presented. This article describes a simulation of the one-phonon coherent inelastic scattering from a lattice model of an arbitrary crystal system. The one-phonon component is characterized by sharp features, determined, for example, by boundaries of the (*Q*, ω) regions where one-phonon scattering is allowed. These features may be identified with the same features apparent in the measured total coherent inelastic cross section, the other components of which (multiphonon or multiple scattering) show no sharp features. The parameters of the model can then be relaxed to improve the fit between model and experiment. This method is of particular interest where no single crystals are available. To test the approach, the poly-CINS has been measured for polycrystalline aluminium using the MARI spectrometer (ISIS), because both lattice dynamical models and measured dispersion curves are available for this material. The models used include a simple Lennard-Jones model fitted to the elastic constants of this material plus a number of embedded atom method force fields. The agreement obtained suggests that the method demonstrated should be effective in developing models for other materials where single-crystal dispersion curves are not available.

## Abstract 2

Presented here is a method by which *Q*-dependent dispersive dynamics may be measured (and used to generate a lattice dynamical model) for polycrystalline samples. This method is analogous to the measurement of dispersion curves for single-crystal samples.

## 1. Introduction

Traditionally, inelastic neutron scattering measurements have involved either incoherent inelastic neutron scattering from polycrystals or coherent inelastic scattering (CINS) from single crystals. The reason that CINS from polycrystals has not been employed to a significant extent in the past is that the process of integrating the scattering intensity over crystallite orientations tends to obscure the useful information that is easily available from the direct measurement of dispersion curves for single crystals measured using a triple-axis spectrometer. However, many important materials can only be obtained in polycrystalline forms, and hence it is of interest to investigate ways of interpreting the coherent inelastic scattering from such samples. One early attempt to do this using coherent inelastic scattering from polycrystalline graphite was made by one of the authors (Ross, 1973 ▶). Since this time, software development and advances in computing power have made it possible (though still demanding) to generate models and to fit them to the data.

There are already several software packages able to calculate the key quantity required in neutron scattering, namely the dynamic structure factor, *S*(*Q*, ω), using the one-phonon eigenvectors as determined *via* density functional theory (DFT) or other methods. Notable examples are the *PHONON* (http://wolf.ifj.edu.pl/phonon/) and *McStas* (Lefmann & Nielsen, 1999 ▶) packages, and efforts have been made to broaden the selection of scattering kernels to include calculated spectral contributions from multiple-phonon scattering, multiple scattering and instrument resolution functions (Willendrup *et al.*, 2004 ▶; *DANSE* project, http://wiki.danse.us) to facilitate direct comparison with the measured data. However, these ‘whole spectrum’ methods have significant limitations. The computational expense associated with the calculation of the eigenvectors of a system is an order *N* ^3^ problem, where *N* is the number of atoms in a basis set describing the lattice; as the number of atoms in a system increases, so the system rapidly becomes too large for the application of *ab initio* methods for the determination of the dynamical matrix. This is further compounded by the reciprocal space sampling requirements for a given model; as the size of the basis set of atoms used to describe the system increases, so the sampling requirements (in terms of *k* points sampled in reciprocal space) also increases. These two factors alone represent an effective limit to the size of the system for which *S*(*Q*, ω) may be modelled using these *ab initio* methods.

In the present work we focus on the calculation of polycrystalline coherent inelastic neutron scattering (poly-CINS) one-phonon cross sections using interatomic potential-based methods, as these are relatively computationally efficient and are widely applicable to many systems (including those with large unit cells) and as the force-constant parameters involved provide a convenient starting point for least-squares optimization of the starting values. Specifically, the method has been implemented within the program *GULP* (Gale & Rohl, 2003 ▶), designed for lattice dynamical calculations based on force field descriptions, *via* a new module known as *Scatter*. However, the approach described could equally well be used in conjunction with DFT packages such as *CASTEP* (Clark *et al.*, 2005 ▶) or *SIESTA* (Sanchez-Portal *et al.*, 1995 ▶), from which phonon eigenvectors may be generated.

The present method differs from a ‘whole spectrum’ approach in that it concerns itself with the identification of prominent one-phonon scattering features appearing in experimental neutron scattering from polycrystals; other contributions (multiphonon, multiple scattering, resolution broadening) would have to be included in the theoretical neutron spectra to provide a full intensity profile for matching to experimental data on a point by point basis. One-phonon processes showing sharp coherent scattering features dominate in the low-*Q* region of (*Q*, ω) space and can be observed experimentally with the best experimental resolution.

An outline of the *Scatter* code has already been published (Roach *et al.*, 2007 ▶), but the methodology behind its application to the interpretation and analysis of poly-CINS is new to this work. It is tested here for the rather simple aluminium system.

The structure of this article is as follows. In §[Sec sec2]2, the theory and methodology of coherent inelastic neutron scattering are described, along with details of the current implementation and the methodology associated with the identification and interpretation of one-phonon scattering features. Also introduced here are four semi-empirical dynamical models of aluminium that are used to compute the dispersion curves and bulk properties for each model. In §[Sec sec3]3, the present experimental measurement using the MARI spectrometer is described. In §[Sec sec4]4, the method developed for the systematic analysis of poly-CINS data is used to compare our experimental data for polycrystalline aluminium with the predictions of the best of the models. The models are also compared with the single-crystal dispersion curve data. Finally, in §[Sec sec5]5, the general applicability of the method to different materials is discussed.

## 2. Methodology

### 2.1. Background theory

The neutron scattering amplitude of a nucleus can have a number of different values, owing to neutron spin and to isotope effects, so the scattering has to be divided into two parts: coherent and incoherent scattering. The coherent part, depending on the average value of the scattering amplitude, contains all the information about the relative position and motion of the nuclei taken in pairs, while the incoherent scattering depends only on the motions of each atom taken independently. As shown by Van Hove (1954 ▶), the resulting cross section can be expressed in terms of the corresponding scattering functions, *S*_coh_(**Q**, ω) and *S*_inc_(**Q**, ω) for the coherent and incoherent scattering cases, respectively. These functions, which depend only on the interactions between the nuclei, define the corresponding double differential scattering cross sections (for materials containing only one element) as follows (Squires, 1978 ▶): 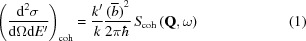
and 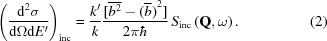
Here, σ_coh_ and σ_inc_ represent the two total cross sections, **Q** is the momentum transfer vector [*Q* = (4π/λ)sinθ being the magnitude of the vector, where θ is half the scattering angle and λ is the wavelength of the incident neutrons], *E*′ is the kinetic energy of a given scattered neutron where the incident energy is *E*, 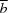 is the average (and 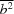 is the mean-square average) of the bound scattering length for the nucleus, *k* and *k*′ are the incident and final wavenumbers, respectively, of the scattered neutron, and ω is the frequency of an excited phonon. The scattering functions, *S*_coh_(**Q**, ω) and S_inc_(**Q**, ω), were originally defined as here for a single species of nucleus. However, for a general non-Bravais lattice (with unit cells containing more than one atom), the terms have to be summed over the atoms in the unit cell, although this results in a scattering function which, in the strictest sense, is not *S*(**Q**, ω) as originally defined. Hence, the effective one-phonon scattering functions for a system with multiple atomic species, as used here, should be written as *S*′_coh/inc_(**Q**, ω), where 
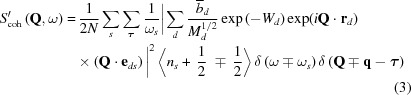
and 
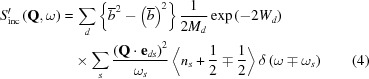
for neutron energy gain [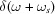 and 〈*n*_s_〉] and energy loss [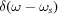 and 〈*n*_s_ + 1〉] of a system, generating a phonon of wavevector **q**. Here *n_s_* is the number of phonons in mode *s* at thermal equilibrium. In equations (3) and (4), *N* is the number of atoms in the unit cell in the (non-Bravais) system and the inner summation is over these atoms, *d*, with mass *M_d_* and a position vector within the unit cell of **r** *_d_*, while *W_d_* is the associated Debye–Waller factor. The outer summations are over **τ**, the reciprocal lattice vector, and *s*, the phonon mode of frequency ω_*s*_, providing a polarization vector **e** *_ds_* for each normal mode, as obtained by solving the dynamical matrix. The reader should be aware that some texts adopt slightly different definitions of the phase factor of the polarization vector. For example, the text by Turchin (1965 ▶) defines the phonon wave at a position **r** *_d_* within the unit cell to have the phase of the travelling wave at that point, whereas Squires (1978 ▶) defines this wave as having a phase relative to that of the travelling wave at the corner of the unit cell. The Turchin version leads to a different general formulation of the coherent inelastic cross section from that given in equation (3). This ‘frame of reference’ difference, whilst being irrelevant for a monatomic lattice, is crucial to the present objective for the case of more than one atom/unit cell. Here the form of the polarization vector used by Turchin will be adopted, as this is the form employed in *GULP*. Hence the corrected form of equation (3) is 
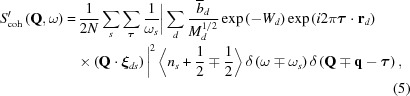
where **ξ** *_ds_* is the polarization vector on the alternative definition given above.

Once the expressions for the neutron scattering functions have been established, the next step is to introduce the means by which the phonon frequency (square root of the eigenvalue) and the polarization vectors (eigenvectors) are obtained for a given phonon wavevector, **q**. The approach, briefly summarized here, follows the standard Born–von Karman method (Born & Huang, 1956 ▶), where forces are described in terms of potentials between pairs of atoms. This method results in the construction of a dynamical matrix of the form 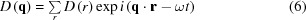
for each of the pairwise vectors, **r**, at reciprocal space wavevector, **q**. The eigenvalues, *ω*(**q**), and eigenvectors, **ξ**, are then obtained from equation (7): 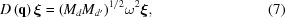
which is solved using 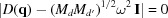 (**I** is the identity matrix) and hence by diagonalizing the resultant matrix. For every **q** vector there is thus a set of 3*d* eigenvalues (and therefore frequencies) and for each eigenvalue a corresponding eigenvector (*i.e.* a set of polarization vectors) for each of the *d* atoms; this results in the notation **ξ** *_ds_* and ω*_s_* for eigenvectors and eigenvalues, respectively.

**Figure 1:**
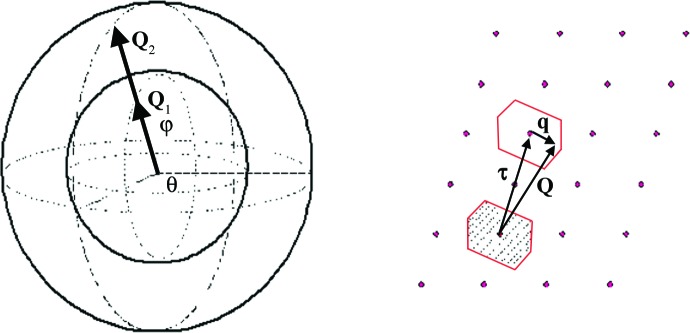
(*a*) The principal poly-CINS reciprocal space sampling method used in the *Scatter* code. (*b*) Selecting the nearest reciprocal lattice point to **Q** provides the reciprocal lattice vector, **τ**, such that **q** remains within the first Brillouin zone (shaded area).

The frequencies and polarization vectors so obtained are then used to calculate the scattering function presented in equation (5). When performing a powder-averaged calculation, it is necessary to sample reciprocal space in a series of concentric shells, each corresponding to a given magnitude of the momentum transfer vector, **Q**, as it is rotated through θ and ϕ. For each magnitude and orientation of **Q**, the program calculates the corresponding values of **q** by selecting the nearest reciprocal lattice point and taking **q** relative to this point. From this value of **q**, the dynamical matrix [equation (6)] is then generated and the corresponding eigenvalues and eigenvectors obtained [see equation (7)]. Fig. 1 ▶ illustrates this sampling method, along with a diagrammatic representation of the **Q** vector relationship with the lattice vector, **τ**, and phonon wavevector, **q**.

The resulting data are then output using a histogram-averaging method, where defined intervals are used to create a mesh of data ‘pixels’; here each pixel corresponds to a set range in the amplitude of **Q** (referred to as *Q*, where *Q* = |**Q**|) and to a set frequency interval. The intensities obtained for this interval in *Q* are sorted into the specified histogram in ω. Assembling such slices for each *Q* yields *S*(*Q*, ω). Resolution broadening of the scattering δ functions in *Q* and ω, Debye–Waller factors may be applied before summation of the intensities, but this is not necessary for present purposes.

### 2.2. Semi-empirical force constant modelling of aluminium

Aluminium is a very well studied material that has attracted attention of late owing to the interest in the use of light metal hydrides for hydrogen storage (Schuth *et al.*, 2004 ▶; Kang *et al.*, 2004 ▶). Results from recent work (Budi *et al.*, 2009 ▶) on the generation of new semi-empirical force constant models for use in cluster calculations has typically been compared against reliable DFT calculations (Pham *et al.*, 2011 ▶) (where comparisons are normally made with zone boundary frequencies), bulk properties (as above), and the density of vibrational states as obtained by both measurement and calculation (Tang *et al.*, 2010 ▶). The simplicity of its crystal structure and the proliferation of these fitting models suggest the use of aluminium as an ideal test material for the methodology presented.

When selecting models for aluminium from the large number of possible variants in the literature the emphasis here has been to test the utility of models in frequent use. For convenience, we will start with a simple Lennard-Jones 12–6 potential (Lennard-Jones & Ingham, 1925 ▶) fitted to the elastic constants (*C*11, *C*22 and *C*44) of aluminium, following the approach suggested by Halicioǧlu & Pound (1975 ▶). Although this type of model is generally considered unsuitable for the study of metallic systems, its very unsuitability (as well as its very low computational cost and the rather general nature of the potential itself) suggested its use as a model for assessing the more heavily parameterized (and considerably more computationally expensive) embedded atom method potentials.

The most successful methodology applied to the semi-empirical modelling of metals, however, is the embedded atom method (EAM) (Daw & Baskes, 1983 ▶), originally developed by Daw and Baskes to study hydrogen embrittlement in nickel and now adapted for use in lattice dynamics and cluster calculations. For this reason, it was decided to test the effectiveness of potentials of this type for predicting the dynamical properties of aluminium and to compare the results with the simplest available potential – the Lennard-Jones (LJ) model, described above.

The EAM models most frequently used in studies of pure and alloyed periodic systems are the Sutton–Chen functional EAM potential (Sutton & Chen, 1990 ▶), the Cleri–Rosato tight binding EAM potential (Cleri & Rosato, 1993 ▶), the Streitz–Mintmire EAM potential (Streitz & Mintmire, 1994 ▶), the Mei–Davenport modified EAM (MEAM) potential (Mei & Davenport, 1992 ▶) and the parameterized EAM (NP-B; Jasper *et al.*, 2005 ▶) potential. Sheng *et al.* have published results for an optimized EAM potential (Sheng *et al.*, 2011 ▶), but as they neglected to include EAM parameters for their model (obtained by fitting to DFT calculations), it has not been practical to include this model in the comparison. Likewise the many-body potential of Mishin *et al.* (1999 ▶), derived from a spline fit to DFT calculations and to bulk data, provided excellent agreement with the measured dispersion curves, but the paper did not include an explicit model parameterization and so this model has also been omitted. Both the Cleri–Rosato and Streitz–Mintmire models were considered for implementation in this study, but as the emphasis here is on the inelastic scattering analysis, these models were neglected for brevity. Hence the three models selected for further analysis are the Sutton–Chen, the Mei–Davenport and the NP-B. Functional forms and explanations of these standard potentials have been omitted from the text, but the expressions and parameters used are given in Table 1 ▶.

### 2.3. Dispersion curves from the semi-empirical models

**Figure 2:**
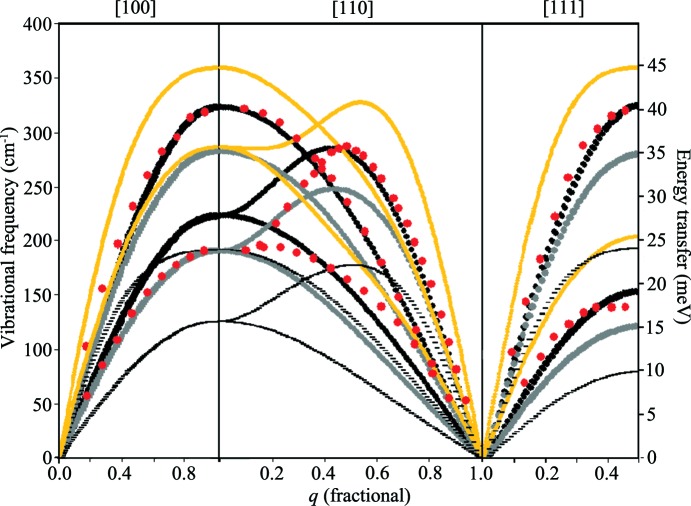
Dispersion curves calculated for the semi-empirical models (with initial parameters) used in this work, compared with experimental triple-axis spectrometer data gathered by Stedman & Nilsson (1966 ▶) at 80 K (red points). Heavy black lines represent the LJ 12–6 model, heavy grey lines the Mei–Davenport EAM, heavy yellow lines the NP-B EAM and thin black lines the Sutton–Chen EAM.

The experimental data presented in this analysis of dispersion curve predictions are taken from the triple-axis neutron spectroscopy study of Stedman & Nilsson (1966 ▶), as presented in Fig. 2 ▶.

As a general preamble, it is worth pointing out the salient features of what is a very well studied system. In the experimental data, the face-centred cubic (f.c.c.) aluminium structure gives rise to the expected form of the dispersion curves in the [100] and [111] directions – doubly degenerate transverse acoustic (TA) modes (as a result of the fourfold and threefold rotational symmetry along these respective axes) and a single longitudinal acoustic (LA) mode. These modes are dominated by the first nearest neighbour interaction (hence the near-sinusoidal form). Thus reasonable agreement for the gradient of the near-linear part of the curve at low |**q**| (the velocity of sound) and the frequency at which the curves cross the zone boundary is sufficient to match the shape of the curves. The observed flattening of the [111] TA curve near the zone boundary is a consequence of contributions to bonding from electron screening and exchange interactions at the Fermi surface (Hafner & Schmuck, 1974 ▶) and, in consequence, cannot be modelled with short-ranged pair potentials. However, the contribution of this feature to the dynamics (and hence to the prediction of bulk properties) is relatively small, and further consideration of this region can be neglected in the present work – especially as it can be modelled with longer-range pair-wise interactions or directly *via* DFT.

The dispersion curves in the [110] direction, along which there is only twofold rotational symmetry, produce an LA mode and two distinct TA modes. The shapes of the TA modes are determined by the second nearest neighbour contributions (even in models that do not explicitly include this interaction); the maximum for the higher-frequency TA mode (found to be displaced from a symmetry point) is determined by the second nearest neighbour contribution, and its accurate positioning requires a model with considerably more near neighbour interactions (eight to ten interaction shells is typical). In aluminium, this maximum is found close to (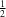 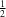
0).

Turning to the predicted dispersion curves, the Lennard-Jones 12–6 potential, fitted to the elastic constants, performs better than all of the EAM potentials in comparison with experiment. It should be noted, however, that this model is specifically targeted at describing the curvature of the experimental (single-crystal) dispersion curves but is not fitted to give zero stress at this geometry. As can be seen in Fig. 2 ▶, it provides excellent agreement with the LA modes in all crystallographic directions. The lack of nearest neighbour interaction terms beyond the first predictably generates only reasonable agreement with the TA modes in the [100] and [110] directions; this clearly demonstrates the need for additional terms for second and further nearest neighbour interactions, as does the inaccurate |**q**| positioning of the [110] TA maximum, although the model does predict the maximum frequency of this mode very well.

It should be noted that the dispersion curves obtained from the EAM models, also shown in Fig. 2 ▶, were not originally compared with the experimental dispersion curve data as their main use has been for studying clusters and associated energetics. Hence, the accurate prediction of the lattice dynamics was less relevant and poor fits are not unexpected. The Sutton–Chen model provides the worst agreement with the experimental dispersion curves, being significantly different from experiment across the entire range of |**q**|, for all three high-symmetry directions, and the prediction of the frequencies at the zone boundaries, most notably the gamma point, provides a poor dynamical description of the aluminium lattice.

The NP-B model, which clearly overestimates the stiffness of the bonding between atoms, produces dispersion curves that are an improvement on those of the Sutton–Chen potential, although the overbinding of the potential produces frequencies for all modes that are considerably higher than the experimental values.

The Mei–Davenport model performs best out of the EAM potentials chosen for this comparison. This model provides a near-perfect agreement for the TA mode in the [100] direction, over the entire range of |**q**|, and gives very reasonable agreement for the LA mode in the same direction, with close agreement in the lower-*q* region (|**q**| < 0.5) and reasonable agreement at the zone boundary (within 20%). The [110] and [111] directions give rise to similarly reasonable agreement to that provided by the LA mode in the [100] direction: going from very good agreement at low *q* (|**q**| < 0.5) to reasonable agreement at the zone boundary in both high-symmetry directions (certainly superior to the other EAM potentials).

There are clearly many more subtleties in the analysis of these dispersion curves. However, the present work seeks to present the equivalency of approach between single-crystal and polycrystalline neutron scattering, and hence further discussion is not relevant to the present objective.

### 2.4. Interpretation of one-phonon polycrystalline coherent *S*(*Q*, ω)

**Figure 3:**
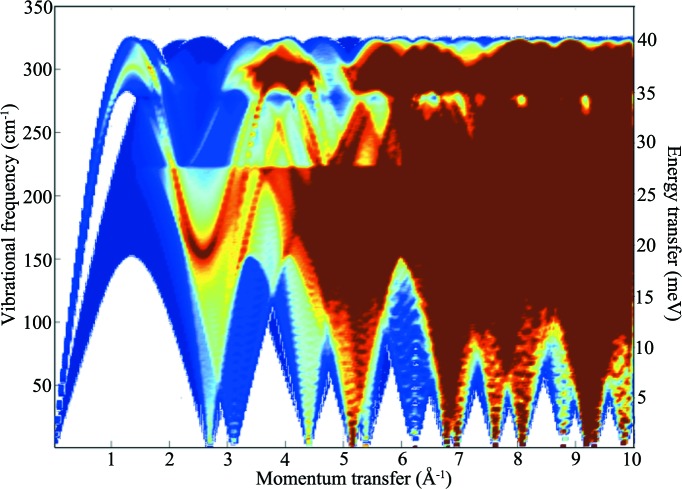
Theoretical polycrystalline *S*′(*Q*, ω) for aluminium, calculated using the LJ 12–6 potential model, for the full range of *Q* (0 ≤ *Q* ≤ 10.0 Å^−1^) and ω (0 < ω < 350 cm ^−1^). The *S*′(*Q*, ω) intensity rises from very low (dark blue) through mid (light blues and yellow) to very high (dark red). White areas denote regions in (*Q*, ω) space where no scattering occurs, whereas dark blue shows low-intensity scattering due to off-symmetry direction (polycrystalline averaged) scattering.

The present approach assumes that the one-phonon coherent scattering process is dominant in the studied system at low *Q*; for the aluminium system this is a reasonable assumption given that aluminium is a strongly coherent scatterer [with a coherent cross section of 1.495 b (1 b = 100 fm^2^), contrasting significantly with the incoherent cross section of 0.0082 b]. The approach also assumes no preferred orientation of crystallites in the sample, as it uses spherical polycrystalline averaging (although intensity changes due to preferred orientation are readily introduced by altering the sampling method): again a reasonable assumption for a polycrystalline cubic system such as aluminium. The resulting contour plot of the poly-CINS intensity for the fitted LJ 12–6 potential is shown in Fig. 3 ▶, which was obtained using the *Scatter* subroutine in the *GULP* program as described above. This poly-CINS plot is a 300 (in *Q*) × 300 (in frequency or energy transfer, *E*_T_) bin data set with 300 angular steps in θ and ϕ, for ranges of 0.0 ≤ *Q* ≤ 10.0 Å^−1^ and 0 ≤ ω ≤ 350 cm^−1^ (0 ≤ *E*_T_ ≤ 43.4 meV). This produces a sampling mesh of 27 million *q* points and samples (*Q*, ω) space in an approximately equivalent *Q* and energy transfer resolution to that provided by time-of-flight instruments such as MARI. The pattern is clearly complex but can be analysed if approached systematically in the light of the experimental or calculated dispersion curves.

**Figure 4:**
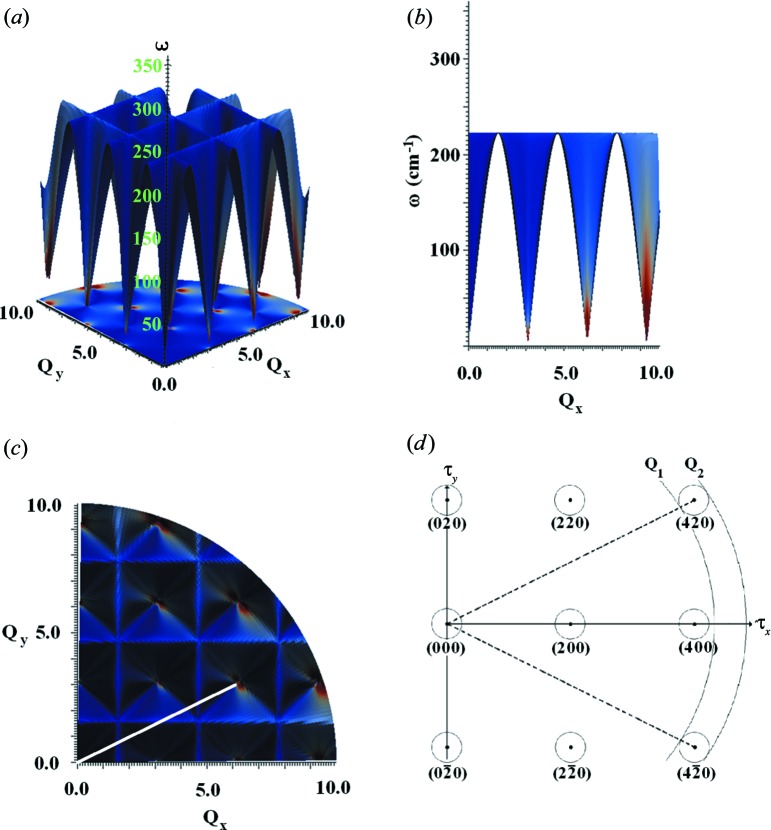
Views of the mode 1 dispersion surface in the *Q_x_Q_y_* plane of the reciprocal lattice of aluminium, using the original Lennard-Jones potential (before fitting has been applied), with scattering intensity superimposed upon the frequency surface as a colour map. (*a*) An isometric projection of the dispersion surface. (*b*) A cross section through the plane from the *Q_x_*-axis perspective. (*c*) The view looking down on the plane. (*d*) A diagrammatic representation of the (*hk*0) plane in aluminium, showing for example the [420] direction (broken line). The two spheres in *Q* show the upper and lower values of *Q* for which one-phonon scattering can occur as a result of the condition |**τ** − **q**| < *Q* < |**τ** + **q**|.

Fig. 4 ▶(*a*) shows the dispersion surface for the first mode (in this case, the longitudinal acoustic mode) overlaid with a colour map that shows the *S*(*Q*, ω) intensity for the plane (*hk*0) over the range of the calculation. Fig. 4 ▶(*b*) takes this projection and rotates it so that the (*h*00) dispersion surface is visible (equivalent to the allowed scattering from the longitudinal mode in the [200] direction), so that the coherence condition is illustrated; from here, one can clearly see the regions of allowed scattering [with the colour map providing *S*(*Q*, *ω*) intensity information – white effectively denotes regions of (*Q*, ω) space where there is no allowed scattering]. From Fig. 4 ▶(*b*), one can clearly see the regions of intense scattering around the Brillouin zone boundaries, as well as the increased intensity corresponding to the 1/ω term in equation (5). Fig. 4 ▶(*c*) shows the same data, as seen from ‘above’, viewing the (*hk*0) plane; this figure rather clearly illustrates the periodic boundary conditions and provides a reference point for Fig. 4 ▶(*d*). The white line denotes the vector from the (000) point in the reciprocal lattice out to the (420) point.

Fig. 4 ▶(*d*) provides an illustration of how it is that some of the sharp features are associated with vectors to more remote reciprocal lattice points. The diagram shows the (*hk*0) plane in reciprocal space and for a particular value of ω that cuts the LA dispersion surfaces close to each ‘allowed’ f.c.c. reflection, out as far as (420). Thus the small circles represent the **q** vectors corresponding to the LA phonons at that value of ω. Because of the coherence condition, the sphere in *Q*, which has to be integrated over direction to yield the intensity expected in the polycrystalline case, has to lie in the range defined by |**τ** − **q**(ω)| < *Q* < |**τ** + **q**(ω)|. Thus we expect to find sharp features for this value of ω where the *Q* sphere touches one of the small circles as illustrated, and this turns out to be along the vectors from the origin to the higher reciprocal lattice points. Noting this, and taking into account the polarization term in the intensity **e** *_ds_*·**Q**, the edge features in the scattering will be pronounced for LA modes because here the phonon displacement vector is parallel to **Q**. Transverse modes, on the other hand, will tend to peak where the *Q* sphere passes through the reciprocal lattice point, as here the displacement vector is parallel to the **Q** vector at the point where the sphere crosses the dispersion surface at right angles (and also passes through the reciprocal lattice point). Because the steps in intensity generally occur along the [*hkl*] directions, they are relatively easy to identify in the integrated one-phonon cross section.

The process presented here is a simple one; reciprocal lattice vectors from the origin of the reciprocal lattice to the first 11 reciprocal lattice points are calculated for the model used in the theoretical generation of *S*′(*Q*, ω) – namely the Lennard-Jones 12–6 model introduced in §[Sec sec4]4. These lines are used to calculate *S*′(*Q*, ω) going through the different (*hkl*) points. As expected, the cross section shows a sharp intensity step where the two spheres touch tangentially (in the longitudinal case) or peak at the (*hkl*) point (transverse case). This provides an initial qualitative appreciation of the features found in the poly-CINS data (as discussed in §[Sec sec3]3). Further clarification, in the form of figures demonstrating this curve projection and overlay process, is provided in the supplementary text^1^ (Fig. S5).

**Figure 5:**
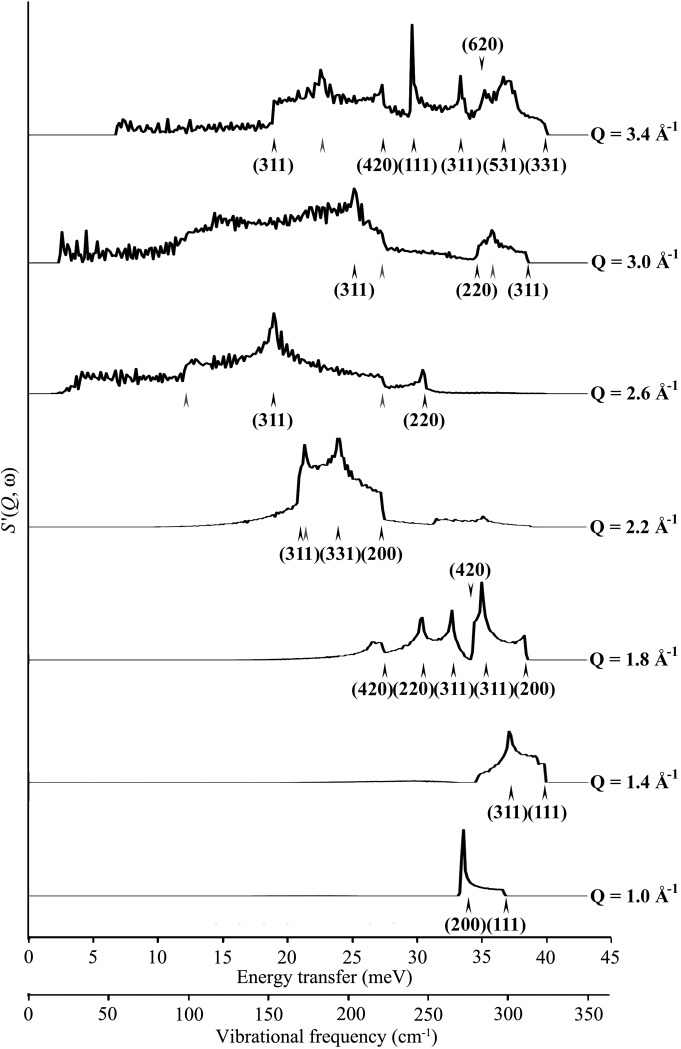
Identification of prominent features in the theoretical poly-CINS data set presented in Fig. 3 ▶. The horizontal axes are energy transfer and vibrational frequency (meV and cm^−1^, respectively) and the vertical axis is *S*′_coh_(*Q*, ω). Major scattering features are labelled for each cut in terms of a **q** point along the direction in the conventional reciprocal lattice [*hkl*]. Black arrows denote an identified feature, with appropriate [*hkl*], in the LJ 12–6 theoretical data and grey arrows denote prominent features that do not correspond to any of the dispersion curves used in this analysis.

The next step in this process is to take cuts through the calculated polycrystalline *S*(*Q*, ω) data for given ω intervals integrated over a narrow band of *Q* values and to associate, where possible, distinct scattering features with individual dispersion surfaces in the symmetry directions selected. In Fig. 5 ▶, cuts of constant *Q* (having a single ‘bin’ width of 0.0334 Å^−1^) have been taken through the theoretical data presented in Fig. 3 ▶. Each cut, which is representative of the *Q* resolution available from a typical high-resolution chopper spectrometer, is then inspected and the most prominent features are compared with the equivalent projections of the dispersion curves. It is observed that the steps in intensity at a given *Q* tend to occur where this cut crosses one of the set of ‘dispersion’ curves calculated along higher-order **τ** directions, as explained above. As noted above, the intensity features observed can be classified in terms of either peaks or coherence edge features; both sets of features are determined by the structure and vibrational characteristics of the material and are governed by equation (4). For the purposes of this work, it is unnecessary to identify every feature in a given cut through the poly-CINS data set, because clearly some will arise where the *Q* sphere touches the dispersion curve away from any symmetry direction.

In this work, coherence edge features have generally been selected for identification, as the use of experimental data requires the summation of adjacent *Q* bins to reduce the statistical error in the measured intensity (owing to the relatively low count rate for a given set of detectors). This results in a ‘smearing’ of both types of sharp feature such that the peak–coherence edge paired features are often the only readily identifiable feature in a given cut through an experimental set. Therein lies the compromise implicit in this approach to the analysis of poly-CINS data; a narrower range of *Q* over which a summation is taken results in sharper *Q*-dependent features, but this in turn reduces the number of measured neutron counts and hence the statistical accuracy of the data. In the extreme, a full summation over the entire range of *Q* sampled by an instrument results in the effective application of the incoherent approximation; here all *Q* dependence is lost and the data collection is reduced to a phonon density of states measurement, whereas a fine cut (as for a single detector), providing the best *Q* resolution, would result in excessively long measurement times to gain sufficient statistics to make an effective comparison with a model. Examples of coherence edge features in Fig. 4 ▶ include the features containing the [111] LA modes at *Q* = 1.0 Å^−1^ and *Q* = 3.4 Å^−1^ and the [200] TA coherence edge at *Q* = 1.8 Å^−1^, for energy transfers of 33.8 meV (273 cm^−1^), 29.8 meV (240 cm^−1^) and 38.6 meV (311 cm^−1^), respectively.

It should be noted that the full intensity of *S*_coh_′(*Q*, ω) at a given (*Q*, ω) value is not exclusively the result of a single mode in a set direction in reciprocal space. Clearly, a large number of **q** values contribute to a given *S*_coh_(*Q*, ω) intensity after averaging over **Q** directions. However, it does seem that many of the sharp features in the scattering functions do arise from the tangential intersection of the sphere in *Q* with a particular dispersion curve.

### 2.5. Bulk properties

Here we present the bulk properties calculated from the models described in the previous section. The values are given in Table 2 ▶, along with experimental values from the literature. Note that the bulk moduli calculated with *GULP* use the Voigt volume derivative approach (Nye, 1957 ▶).

As shown in Table 2 ▶, the potentials all perform adequately when compared with experiment; the single exception to this is the binding energy predicted by the LJ 12–6 pair potential. As is to be expected, this two-parameter LJ model is unable to fit the binding energy, lattice parameter and mechanical properties simultaneously. Because of its pairwise nature, the LJ 12–6 potential cannot capture the Cauchy violation between the elastic constants *C*_12_ and *C*_44_, unlike the EAM models. However, this simple model, when fitted to the elastic constants, provides good agreement across a range of other bulk properties, in particular, the bulk moduli; the model predicts Young’s modulus especially well compared with the other models. It also performs adequately for the bulk and shear moduli, although both the NP-B and Sutton–Chen potentials do better, with the Mei–Davenport outperforming the Lennard-Jones by a considerable margin. However, the Lennard-Jones model performs very well in the prediction of Poisson’s ratio; the EAM models all predict values that are significantly different from experiment. No particular conclusions should be drawn from the agreement with elastic constants as these were used to parameterize the model. Thus, on the whole, the simple LJ 12–6 model performs remarkably well with respect to the curvature of the potential energy surface; the Mei–Davenport model generally matches, or slightly exceeds, its performance for most of the bulk properties compared here, while the Sutton–Chen and NP-B potentials generally have a worse overall performance in this regard.

When comparing the potential models it is important to consider the relative computational expense, given that calculations of poly-CINS will require many second-derivative evaluations. As an example, the LJ 12–6 model completes a standard sampling of (*Q*, ω) space, as described in §[Sec sec2.4]2.4, about 300 times faster than the equivalent EAM calculations. To be precise, the LJ 12–6 model takes around 150 s on a 8-core, 3 GHz Xeon workstation, whereas the EAM potentials average around 45 000 s on the same workstation for the identical sampling and phonon calculations; it is thus clear that the LJ 12–6 model can be useful as a tool for generating the bulk properties in systems with large numbers of atoms in the unit cell, although its poor binding energy and more limited transferability to other environments, such as cluster calculations, limit its broader utility.

## 3. Experimental

In order to collect data across a full range of momentum and energy transfers for aluminium (necessary to investigate the *Q* dependence of the vibrational modes in a poly-CINS experiment), the most appropriate instrument type is a direct geometry time-of-flight chopper spectrometer. This type of instrument [of which the MARI spectrometer (Taylor *et al.*, 1991 ▶) situated at the ISIS facility, UK, is a distinguished example] is designed to sample (*Q*, ω) space by means of a fixed incident neutron energy, where the scattered neutrons are measured using a large detector bank so that energy and momentum transfer can be recorded independently.

**Figure 6:**
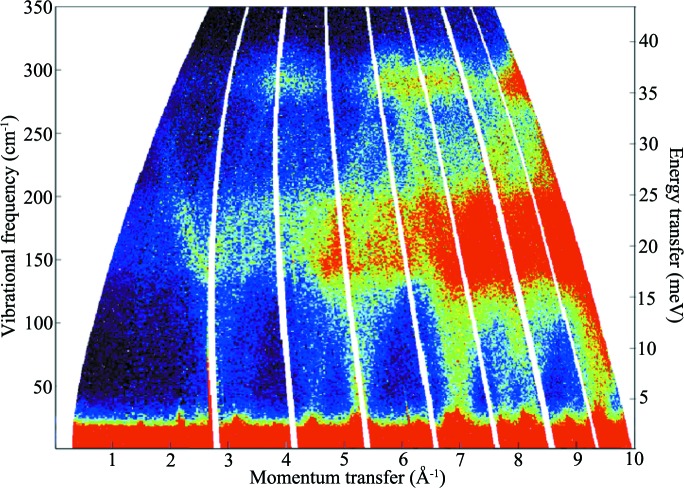
Experimental polycrystalline *S*′(*Q*, ω) for aluminium at 10 K obtained on the MARI spectrometer, ISIS, for the full range in neutron energy loss of *Q* (0 ≤ *Q* ≤ 10.0 Å^−1^) and ω (0 ≤ ω ≤ 350 cm ^−1^). The *S*′(*Q*, ω) intensity rises from very low (dark blue) through mid (yellow) to very high (dark red). White areas denote regions in (*Q*, ω) space where there is no detector coverage.

Data were gathered on this spectrometer over a period of 22 h, using the proton current convention for a total proton current on target of 3600 µA h for ISIS TS1. The sample was a 94 g polycrystalline sample of 99.999% purity aluminium in pellet form (manufactured by Goodfellow Cambridge Ltd) at a temperature of 10 K. The fixed incident energy was 58.8 meV, which provided a sampling of (*Q*, ω) space for 0 ≤ *Q* ≤ 10.0 Å^−1^ and 0 ≤ ω ≤ 350 cm ^−1^ (0 ≤ *E*_T_ ≤ 43.4 meV) in the characteristic ‘bishop’s mitre’ configuration obtained from the detector coverage in neutron energy loss. Although the instrument also collects data in neutron energy gain, neutron energy loss was preferred (and the experiment was performed at low temperatures). This approach more fully samples the region of (*Q*, ω) space of interest, because neutron energy gain is limited to energy transfers ∼*kT*, *i.e.* a few meV at low temperature, where there will be little intensity for energy gain above this. The data have been corrected for detector efficiency, and all other pre-processing and data reduction from raw time-of-flight data to *S*′(*Q*, ω) were accomplished using the *MANTID* suite of neutron scattering instrumentation software (http://download.mantidproject.org/). The resulting data set is shown, with a 10% maximum intensity cutoff applied to avoid domination of the contour plot by the elastic line, in Fig. 6 ▶.

Applying the neutron scattering theory described in the previous section, it is possible to gain a good understanding of the sharp features present in such data sets owing to one-phonon scattering – generally due to the sphere in *Q* touching and crossing particular dispersion surfaces. Multiple-phonon terms, which will dominate at larger energy and momentum transfers, are considerably smoothed relative to the one-phonon terms, as are multiple-scattering effects.

Common to all such coherent polycrystalline *S*_coh_′(*Q*, ω) plots, the one-phonon scattering functions as given by equation (5) (as a function of *Q*) yield scattering intensities that, on average, increase as a function of ∼*Q* ^2^ until attenuated by the increasing contribution of the Debye–Waller factor, exp(−*W_d_*) at higher *Q*.

Upon visual inspection, the first and most obvious features of the data in Fig. 6 ▶ are the ‘arches’ of very intense scattering associated with the LA dispersion curves, corresponding to the superposition of the single crystallographic directions consistent with the dispersion curves of a single crystal. These features, which dominate the scattering intensities in the experimental data, arise from the fact that, for a particular energy transfer, ℏω, the coherence condition implies that |**τ** − **q**(ω)| < *Q* < |**τ** + **q**(ω)|, where **q** is taken along the phonon branches in high-symmetry directions in the Brillouin zone (in particular, for aluminium, the [200], [220] and [111] directions). As seen on closer inspection, the lower-symmetry directions defined by vectors from the origin to higher-order Bragg peaks are also significant. Otherwise, given the relative complexity of these data, identifying which feature belongs to a given vibrational mode is challenging, especially when one considers systems with more than one atom per unit cell or materials with noncubic symmetry. However, it is clear that many of the dominant features in a given poly-CINS data set, and, in particular, the simplest case of a face-centred cubic metal with a single elemental basis, such as aluminium, are those defined by the modes in the major crystallographic directions defined by the **τ** vectors of the reciprocal lattice projected out into **Q**. This observation informs much of the analysis provided in §[Sec sec4]4, as the approach taken to the analysis of the data relies upon the identification of scattering features using projections of dispersion curves onto momentum transfer, **Q**. Thus, the ‘arches’, corresponding to the LA modes in the [111], [200] and [220] crystallographic directions as they return to the elastic line (zero energy transfer) at 2.68, 3.1 and 4.4 Å^−1^ are clearly visible, with further multiples of these at 6.2, 8.8 and 5.34 Å^−1^ and so on. These features are accompanied by the modes returning to zero energy transfer obtained from the vectors to the higher reciprocal lattice points for the f.c.c. lattice, such as the [311], [331], [420], [422], [511], [531], [442] and [620] directions in the conventional (Cartesian) reciprocal lattice directions.

The other key features that are clearly identifiable from this dispersion curve comparison are the maximum energies of the LA and TA modes that give rise to cutoff features in the poly-CINS scattering associated with abrupt intensity changes that are invariant in *Q* but seen as ‘bands’ of intense scattering over the range of energy transfer. Beginning at low *Q*, the first such features are those due to the maxima of the LA modes of the [111] and [100] dispersion curves at *Q* = 1.34 Å^−1^ and *Q* = 1.56 Å^−1^, respectively, at vibrational frequencies (energy transfers) of 325 cm^−1^ (40.3 meV) and 322 cm^−1^ (39.9 meV) that give rise to the high-frequency limit of the scattering. (Note that if **Q** is parallel to **q**, as in the first zone, the condition leads to the longitudinal modes having maximum intensity and the transverse modes zero intensity.)

## 4. Analysis of coherent inelastic neutron scattering from polycrystalline aluminium

In this section, the methodology presented in §[Sec sec2.4]2.4 is applied to the specific task of identifying and exploiting the poly-CINS data from polycrystalline aluminium. Given that data sets obtained from powder samples can be thought of as a multitude of dispersion curves [deriving from every possible direction in (*Q*, ω) space] superimposed upon each other, great care should be taken in identifying any given feature as belonging to a particular phonon branch. This is particularly so, given that it was found that many of the most intense sharp features in the poly-CINS data set arise where the sphere in *Q* crosses the dispersion surface in nonsymmetry directions. However, by extending the analysis to include crystallographic directions in reciprocal space other than the highest symmetry directions, many of these features can be identified. This then allows the experimentalist to identify specific features and use the frequencies of these features in a fitting process to generate theoretical models that match these features. Given that the intensities of poly-CINS features are directly related to the eigenvectors for the motions of planes of atoms, this method presents an excellent means of generating physically useful models that can predict bulk properties well.

The approach taken in this work has been to identify the dominant features of the aluminium *S*′(*Q*, ω) spectrum using the simple LJ 12–6 model. Once the general features of the plots are identified, it becomes relatively straightforward to extract effective dispersive feature information in terms of more traditional dispersion curves, which can then be used to create a set of *k* points for use in a fitting process, as described in §[Sec sec4.1]4.1. This method is applied to the experimental data set to illustrate the utility and limitations associated with the analysis of experimentally obtained data. In §[Sec sec4.2]4.2, the same approach is applied to a comparison of the ‘best’ of the MEAM models, the Mei–Davenport model, with the theoretical neutron spectra; dispersion curves and bulk properties are then recalculated for both models fitted to experimental data. It should be noted that no resolution correction has been added to the theoretical data, as this makes it easier to identify the coherence edges, which are then linked to the sloping edge features in the experimental data (see §[Sec sec4.1]4.1).

### 4.1. Comparison of experimental Al poly-CINS *S*′(*Q*, ω) data with model predictions

**Figure 7:**
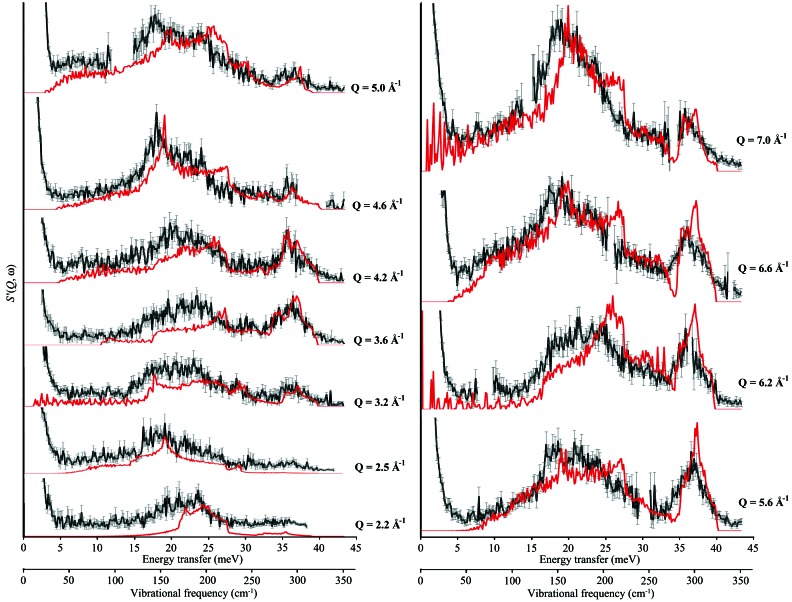
Selected cuts at constant *Q* through the experimental *S*′(*Q*, ω) (black line) for aluminium at 10 K obtained on the MARI spectrometer, ISIS, from *Q* = 1.0 Å^−1^ to *Q* = 7.0 Å^−1^. The horizontal axes are energy transfer and vibrational frequency (meV and cm^−1^ respectively) and the vertical axis is *S*′_coh_(*Q*, ω). The width of cut is 0.067 A^−1^ and the corresponding cut through the LJ 12–6 theoretical data (fitted to elastic constants) is given as the red line. All intensities are to scale.

As noted above, the full intensity of *S*_coh_′(*Q*, ω) at a given (*Q*, ω) value is a superposition of the scattering seen in all directions. That is why it is necessary to concentrate on the identification of particular sharp features for the comparison of experiment and theory. Once *S*_coh_′(*Q*, ω) features are identified as being associated with a given cut in reciprocal space, the next step is to approach the experimental *S*(*Q*, ω) data and attempt to identify equivalent features. In order to facilitate the comparison, both experimental and theoretical cuts (the LJ model) through the data sets at fixed *Q* values may be superimposed as in Fig. 7 ▶.

We note here that there are gaps in the detector coverage on the instrument and these affect the choice of constant *Q* cuts. Hence a new set of cuts, chosen to pass through the sharpest, most intense one-phonon features with a minimum of gaps due to missing detectors, were selected: cuts were made through both theoretical and experimental data sets for a bin width of 0.0667 Å^−1^ (median value ±0.03335 Å^−1^) for median values of *Q* of 2.2, 2.5, 3.2, 3.6, 4.2, 4.6, 5.0, 5.6, 6.2, 6.6 and 7.0 Å^−1^. The resulting comparisons are shown in Fig. 7 ▶.

Although there are significant differences in both the intensity profiles and the positions of sharp features on the energy transfer scale, the general *S*_coh_′(*Q*, ω) profile is in reasonable agreement: sufficiently so to relate features in the theoretical and experimental profiles.

From these comparisons, 31 *S*_coh_′(*Q*, ω) features could be reasonably assigned to one of the 11 reciprocal space directions used for this treatment. In order to reduce the likelihood of assignment errors in this process, a Python script was written to automate the selection process somewhat; each potential matchable feature in the experimental data (whether peak or coherence edge) was selected directly from the relevant data slice and the corresponding energy transfer for the feature was recorded; these values were then used as sort parameters. This sort generated a list of the nearest theoretical dispersion surfaces in our set of [*hkl*] directions, and only surfaces that passed through the feature within 1 cm^−1^ (Δω) were considered as potential matches. Depending on the relative sharpness of the scattering feature, some features of less than 1 cm^−1^ were discarded as being insufficiently clear to match. Each sort then provided the dispersion curve label, the **q** point (in terms of fractional reciprocal lattice vector) and the mode number (using the mode identification in *GULP*) for use in model fitting, assuming that the Δω condition was met and the feature was sufficiently distinct in both theoretical and experimental data sets.

This process is clearly amenable to automated feature identification *via* a mathematical optimization formalism. However, for this initial work, the same process as is used for mode assignment in other spectroscopy has been used and only clearly distinct features have been selected for potential comparison to experimental data.

**Figure 8:**
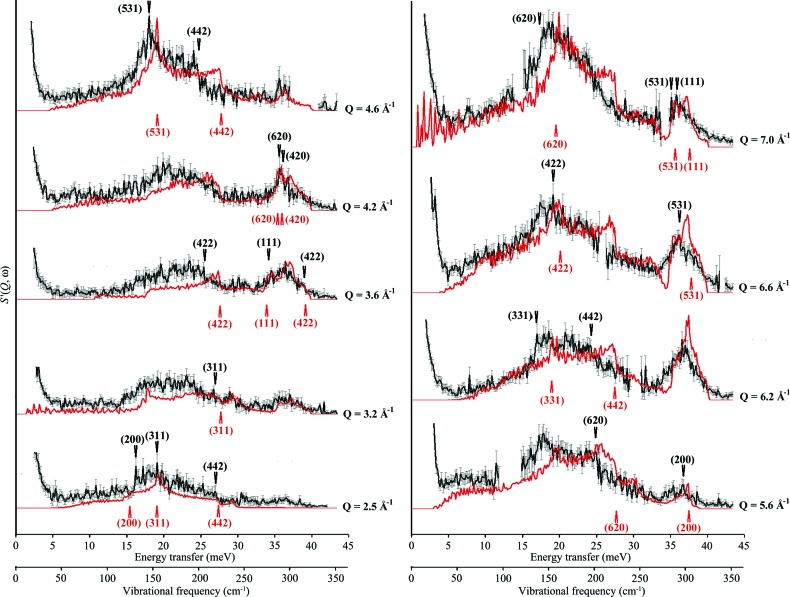
Four constant *Q* cuts through Fig. 3 ▶ (red line) and Fig. 7 ▶ (black line) for a *Q* bin of width of 0.067 Å^−1^. The horizontal axes are energy transfer and vibrational frequency (meV and cm^1^, respectively) and the vertical axis is *S*′_coh_(*Q*, ω). Major scattering features are labelled for each cut in terms of a point along the direction in the conventional reciprocal lattice [*hkl*]. Red arrows (below spectra) denote an identified feature, with appropriate [*hkl*], in the LJ 12–6 theoretical data and black arrows (above spectra) the equivalent feature in the MARI data. Relative intensities between cuts are not to scale, for clarity of presentation.

With these criteria, of the 31 identified features in 11 cuts through the Lennard-Jones-derived data, only 20 could be unambiguously assigned to the experimental data. The labelling is provided in Fig. 8 ▶ for these 20 features, where the arrows are labelled with the relevant direction in **q** space. The upper values (black) refer to the experimental data and the lower values (red) to the model. To aid in the visualization of these points, Fig. S5 (in the supplementary text) shows how the sampling of (*Q*, ω) space has been accomplished, by projecting the points labelled in Fig. 8 ▶ onto the *Q* scale used for Figs. 3 ▶ and 6 ▶.

The final step in the analysis process involved fitting two of the models (LJ and Mei–Davenport) to these (**q**, ω) points using *GULP*’s internal least-squares minimization routine. In this procedure, the (**q**, ω) points were input as observables and the optimal parameters were obtained for both models from a variety of initial values to ensure that a reasonable global minimum was obtained. For the Mei–Davenport model, the EAM density terms were not fitted, although the lattice was allowed to relax in one fit and fixed in the other to explore the effect of fixing the lattice in such a model.

### 4.2. Results from the fitting of poly-CINS data to two models for aluminium

The fitting process proved very successful for the case of the Lennard-Jones potential model, and a global minimum was found with values of *A* and *B* of 28273.896 eV A^−12^ and 46.590 eV A^−6^, respectively, keeping the lattice constant fixed. The fitting process applied to the Mei–Davenport model, whilst successful, was considerably more methodologically dubious as the parameter space for the process is considerably larger and, as this work is not specifically targeted at producing a more physically accurate MEAM model, little effort was spent on ensuring that a true global empirical minimum was reached. However, as the EAM density parameters were kept fixed, *i.e.* were not included as fitting parameters, the fit resulted in changes to the *E*_c_, α, β and γ parameters (with new values of 3.335, 4.57, 7.047 and 11.326 eV, respectively) in the EAM functional part of the potential, with *ϕ*_0_ and δ changing to 0.1330 and 7.3866 eV, respectively. The final parameterization of each model is presented in Table 3 ▶.

**Figure 9:**
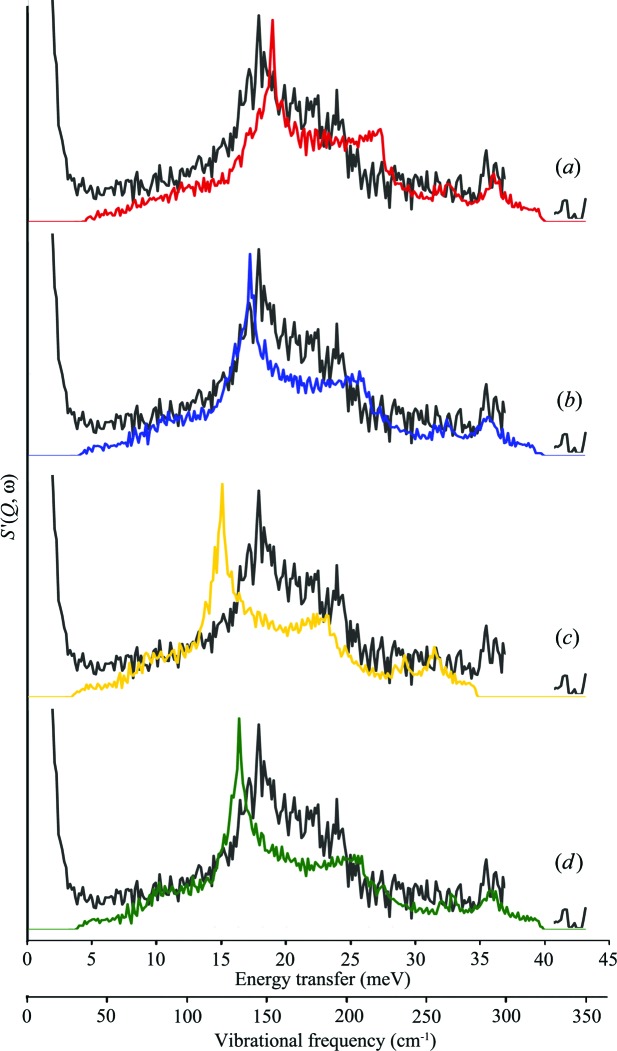
Comparison of four theoretical models, using the constant *Q* cut (width of 0.067 Å^−1^) for *Q* = 4.6 Å^−1^ from Fig. 8 ▶, with experimental data for aluminium at 10 K obtained on the MARI spectrometer, ISIS (grey line). (*a*) The original LJ 12–6 model, (*b*) the LJ 12–6 model fitted from MARI experimental data, (*c*) the original Mei–Davenport model and (*d*) the Mei–Davenport model fitted from MARI experimental data. The horizontal axes are energy transfer and vibrational frequency (meV and cm^−1^, respectively) and the vertical axis is *S*′_coh_(*Q*, ω).

Thus, in Fig. 9 ▶, the initial and final (fitted) versions of the Mei–Davenport and Lennard-Jones models were used to generate *S*_coh_′(*Q*, ω) data sets equivalent to those found in Fig. 3 ▶, and constant *Q* cuts were taken through these data sets, following the process described in the previous section. The figure presents a representative example [for median *Q* = 4.6 Å^−1^, with a cut width of 0.0667 Å^−1^ (median value ± 0.0334 Å^−1^)] of these data comparisons; more have been provided in the supplementary text. As can be seen, the key features being tracked are the peak corresponding to the [531] dispersion curve at 145 cm^−1^ (18.0 meV) and the coherence edge feature corresponding to the [422] dispersion curve at 200 cm^−1^ (24.8 meV) in the experimental data. Curves (*a*) and (*c*) show the theoretical *S*_coh_′(*Q*, ω) generated from the initial versions of the models and clearly illustrate the better agreement with experiment provided by the LJ model. Curves (*b*) and (*d*) are the equivalent *S*_coh_′(*Q*, ω) data generated after the fitting procedure It is clear that both models show significant improvement; in particular, the peak corresponding to the [531] direction shows much improved agreement with the experimental data. This is not surprising given that the features discussed were used as observables in the fitting process. However, selection of other cuts also produces features that are in better agreement with experiment (see supporting text for additional examples).

At this point in the analysis, it becomes clear that the present process lends itself very naturally to an iterative approach to the fitting process; although the present treatment deals with only a single ‘run through’ of the method, the most sensible means of improving fits [and hence the quality of the model generating *S*_coh_′(*Q*, ω)] would be to take the outputs of the current method and, rather than moving straight to bulk properties and dispersion curves, to re-apply the process (probably several times) by taking cuts through the data set generated by the new model(s) and running through the feature identification and the fitting steps again. This approach, which would resemble a profile refinement process as used for diffraction data (in effect, inelastic profile refinement), would minimize the differences between the theoretical and experimental *S*_coh_′(*Q*, ω); work is in progress to demonstrate this approach.

**Figure 10:**
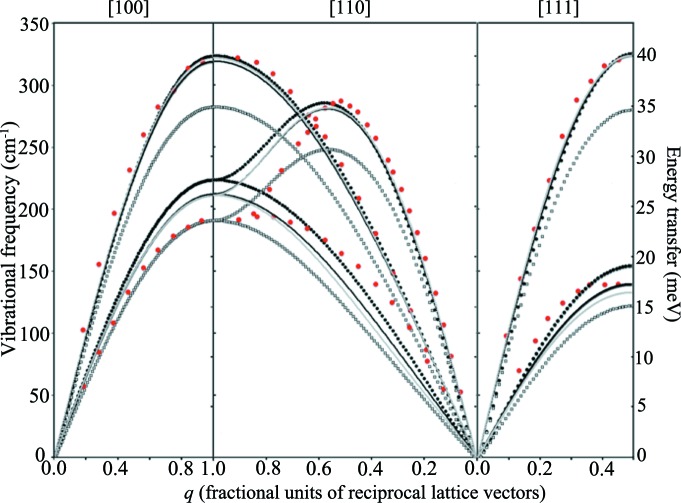
Dispersion curves calculated for the semi-empirical models fitted to experimental data in this work, compared with the experimental triple-axis spectrometer data (red points) previously presented in Fig. 3 ▶. Heavy black lines are the original LJ 12–6 model, thin black lines the fitted LJ model, thick grey lines the original Mei–Davenport model and thin grey lines the fitted Mei–Davenport model.

Once the fitted models have been inspected for agreement with experiment, the final stage in the analysis is that of generating bulk properties and dispersion curves for the original and fitted models. Fig. 10 ▶ presents the dispersion curves for the two versions of the Lennard-Jones and of the Mei–Davenport models compared with the single-crystal dispersion curve data of Stedman & Nilsson. Rather encouragingly, both fitted potential models generate dispersion curves that are very similar, illustrating that the sampling of reciprocal space is consistent for both models. Both of the fitted models also produce dispersion curves that are in better agreement with the experimental curves, after allowing for the difference in temperature: the Stedman & Nilsson data were taken at 80 K, whereas the models generated are for data taken at 10 K. The higher temperatures will result in slightly softer modes and hence lower maximum frequencies at the zone boundaries. Of course, none of the models effectively reproduce the curvature of the TA modes in the [110] direction: both models have very short cutoff distances for the interactions (first nearest neighbour for the LJ model and fourth nearest neighbour for the Mei–Davenport model), which will significantly influence the curvature of the dispersion curves in this region. Indeed, the work by Gilat & Nicklow (1966 ▶) suggests that effective Born–von Karman force constant models in metals such as aluminium are sensitive to nearest neighbour interactions out to at least the eighth nearest neighbour distance. However, both models do show an improved agreement with experiment (both for the single-crystal values and for the data reported in the present work).

Finally, the bulk properties of aluminium were calculated for both fitted models. The results are presented in Table 4 ▶. The fitted Lennard-Jones model, which produces improved dispersion curves, nevertheless performs somewhat less well than the original model as far as the bulk properties are concerned. This is unsurprising given that the initial model was fitted to experimental elastic constants, but changes to the force constants *A* and *B* are relatively small. The bulk property calculations for the Mei–Davenport model were handled in two ways; the first was a straight fixed-lattice calculation where the potential generated very good agreement with experimental data; in the case of binding energy (not strictly valid for fixed-lattice calculations), Young’s and shear moduli, and elastic constant predictions, the fitted model outperforms the original model and generates predicted bulk properties consistently closer to experimental values. With the constraint of a fixed lattice removed, the model fitted to the *S*_coh_′(*Q*, ω) data produced a second set of predicted bulk properties (in parentheses in Table 4 ▶). Although this results in a relaxation of the lattice, giving rise to a lattice constant that is significantly too large (4.17 Å, rather than the experimental value of 4.05 Å), the model performs much better than either the Lennard-Jones (fitted) or the original Mei–Davenport model in terms of predicted binding energy and the other bulk properties, especially the elastic moduli (Young’s, bulk and shear). We have demonstrated that the results can be markedly improved simply by expanding the cutoff of the LJ 12–6 model to 12.0 Å. This results in a model that provides broadly similar agreement (in terms of bulk values) to that of the original LJ model (fitted from elastic constants), yet requires no adjustment of the lattice constants (*i.e*. the model predicts a lattice spacing of 4.05 Å when allowed to relax). The resultant values of the parameters are included in Table 3 ▶ but further discussion is postponed.

### 4.3. Discussion on the extension of the method to other systems

As has been stated earlier, aluminium was chosen to illustrate the methodology for this work because it has the simplest possible crystalline structure as well as being a very well understood material in terms of both bulk properties and dynamics. Given the complexity of polycrystalline data sets, aluminium represents the simplest possible case to apply in terms of both basis size and symmetry, yet for this method to be generally useful, it should be extensible to materials with polyatomic basis sets and should also provide some demonstrable advantages to the current method of analysing powder spectra – namely the incoherent approximation.

Currently, the standard approach to assigning vibrational frequencies for polycrystalline coherent scattering systems is *via* the so-called incoherent approximation, a detailed treatment of which can be found elsewhere (Mitchell *et al.*, 2005 ▶; Kearley, 1995 ▶). In this approach, the experimental data are integrated over the full angular range of detectors, which provides greatly improved statistics at the cost of obscuring all explicit *Q* dependence in the resulting two-dimensional (frequency *versus* integrated intensity) data sets. The general method effectively treats the resulting data as a means of experimentally determining the phonon density of states, *g*(ω). These data are then compared, with minimal processing, with a gamma point calculation [typically using DFT and software such as *PHONON* or *a-CLIMAX* (Ramirez-Cuesta, 2004 ▶) to post-process the force constant outputs into simulated inelastic neutron intensity data], and some implicit *Q* dependence is then inferred from relative intensities or the intensity profile as a whole. As an analysis method, it has much to recommend it: the method is very computationally efficient (a gamma point calculation is relatively computationally inexpensive and can be performed on a desktop workstation in very little time for small-unit-cell systems) and, as has been mentioned, there are a number of freely available support software packages available to assist in this analysis. However, it is clear that there are several issues with this approach, the most significant being that explicit *Q* dependence of the vibrational modes is lost entirely; as one is dealing with the density of vibrational states, one may not track the curvature of individual modes. Indeed, before the computational resources became available for routine DFT calculations, much of the most detailed inelastic scattering simulation and fitting used semi-empirical models and carefully considered correction factors (Egelstaff & Poole, 1969 ▶) to properly weight the integrated intensities. The scattering data were plotted as a function of *Q* ^2^ at a given angle, and the high-*Q* data were extrapolated back to yield a gradient at small *Q* ^2^, where the actual data shows large fluctuations due to coherent terms. This would result in semi-empirical force constant fits that could be very difficult to interpret and assign properly, given the sensitivity of the fits to the relative intensity profiles (Kearley, 1995 ▶).

After the advent of the easily accessible computational resources required to directly generate intensities using DFT, the emphasis shifted to using the aforementioned DFT direct simulation approach, typically compared with directly integrated *g*(ω). However, this approach directly integrates the scattering over observed values of *Q* at a given energy and assumes that the coherent scattering averages out. Our calculations show that there are significant differences between the actual density of states obtained from *GULP* and the above quantity obtained from the *GULP* poly-CINS output. The most notable one is that of intensity differences between the strict incoherent density of states, especially at low *Q*, where contributions from the coherent scattering in this region produce a ‘summed up over *Q*/angles’ profile for the incoherent approximation that significantly deviates from the density of states. There are also issues associated with fitting an improved model: DFT calculations do not, in general, provide precise matches to INS data sets, and the scaling approach (Mitchell *et al.*, 2005 ▶) used to assign modes unambiguously can obscure the quality of a given calculation. In any case, DFT outputs cannot be ‘fitted’ to provide functional models for *T* > 0 K systems with ease. The poly-CINS method does not suffer from this limitation (although DFT inputs can be used to provide an initial starting point for finite temperature calculations), as the *Q* dependence of individual modes can be identified and tracked, without resort to either complex correction factors or scaling of frequencies.

As the size of the basis set increases from the monatomic basis used for this work, other features in the scattering data will become relevant to discuss – most notably the scattering from optic modes (as aluminium is monatomic, it does not possess optic modes). Although this work has restricted itself to the discussion of the dynamics of the aluminium system, and by definition scattering profiles derived from acoustic modes, the authors have measured a number of polyatomic systems (with lower symmetries than cubic aluminium) using this approach, most notably graphite (Roach, 2006 ▶; Roach, Heuser *et al.*, 2013 ▶; Roach, Parker *et al.*, 2013 ▶), MgD_2_ (Buckley *et al.*, 2013 ▶) and C_60_ (Roach, 2006 ▶; Roach, Heuser *et al.*, 2013 ▶; Roach, Parker *et al.*, 2013 ▶). In all these cases, optic modes generate the same coherence edge and peak features (they are still subject to the same selection condition as acoustic modes) and can be measured and identified, although these can be more difficult to distinguish than in the present case, requiring extremely long data collection runs on high-intensity instruments to provide sufficiently high count rates to distinguish these features from noise. This is especially so in the case of very large systems and systems with relatively ‘flat’ optic mode branches (such as C_60_), which require correspondingly high resolution sampling in the calculation of the poly-CINS intensities. Lower-symmetry systems also appear to pose no particular problem, providing the sampling of reciprocal space is sufficiently tight.

Finally, zone boundary effects can add to the variation of intensities, and one might speculate that this issue might affect the identification of edge and peak features where scattering close to the zone edge is very much more intense (see Fig. 4 ▶ *b* and comments in §[Sec sec2.4]2.4); indeed, it is very likely that a number of the most intense features in Figs. 7 ▶ and 8 ▶ are due to the orientational averaging of this effect. This is, however, more likely to be an issue with samples with crystallites of higher-symmetry materials (such as aluminium), as the Brillouin zone path is very simple and these effects will superimpose in the orientational averaging much more obviously. With lower-symmetry crystallites, this would probably average out into a smoother intensity profile. However, it is important to check on this. Fig. S5 in the supplementary text provides a convenient method of checking for bias introduced as a result of this effect, as well as a means of checking other sampling bias (TA rather than LA, for instance, or zone centre *versus* zone edge). In the case of aluminium, and the sampling and feature selection for this work, it appears that the sampling is evenly split between zone edge and other regions. It is quite clear that some coherence edge features are more pronounced near the zone edges, but it is also clear (from Figs. 8 ▶ and S5) that scattering beyond these areas is sufficiently intense to identify these features away from the zone edge.

## 5. Conclusions

In the present paper we describe the structure of an extension to the *GULP* program, which calculates the polycrystalline coherent inelastic neutron scattering directly from a dynamical matrix for the crystal. This model for *S*_coh_′(*Q*, *ω*) can be compared directly with experimental measurements made using an inelastic neutron spectrometer, either as a complete contour plot or in terms of profiles along observed loci in (*Q*, ω) space. The method has been applied to aluminium as a simple example of a crystalline material for which dispersion curve data are available; this provides definitive corroboration of the methodology. Three popular semi-empirical models have been simulated, along with a simple model generated for the purpose of method checking. It was found that these models differ significantly from each other in terms of the dispersion curves and the bulk properties predicted by each. The best performing of these four models (the Lennard-Jones and the Mei–Davenport) were then used to identify and analyse the polycrystalline coherent inelastic neutron scattering spectrum from a polycrystalline aluminium sample. Cuts were used through the data to identify dispersive features that were then used as fitting observables in a least-squares fit of the model to the neutron data.

The method described, which relies on the analyst to identify scattering features in the neutron data that correspond to single-crystal dispersion curves in a polycrystal, has proven to be effective in the simple case of aluminium. The strength of the method is that it does not require full sampling of (*Q*, ω) space – even along the locus of a cut through experimental data – to generate fitting observables. Rather it applies an understanding of the ‘rules’ behind coherent inelastic scattering to identify scattering features that coincide with dispersion curves in single-crystal samples, and then uses these points in (*Q*, ω) space to generate individual **q** points for fitting using lattice dynamics codes. Furthermore, although fits to experimental data can be improved with the addition of computationally calculated two-phonon and multiphonon scattering contributions, dynamic Debye–Waller factor contributions, multiple scattering corrections, and other (instrument-specific) contributions, none of these are necessary to identify the majority of one-phonon edges in a polycrystalline sample. This in turn means that the computational costs for a given analysis are orders of magnitude less than would be required for a full calculation using a total scattering approach. As one increases the size of a given unit cell, the computational expense of the full calculation rapidly becomes prohibitive and the required sampling of reciprocal space itself becomes a limiting factor; even simple systems with unit cells containing less than 20 or so atoms require significant computational resources for such calculations. Work is underway on the extension of this method to systems with larger unit cells. With regard to the complementarity of the poly-CINS method with incoherent inelastic neutron scattering, it is worth pointing out that, for systems dominated by incoherent scattering that are not suitable for isotope substitution with coherently scattering nuclei, the poly-CINS method would provide little additional useful information and so mode assignment *via* incoherent scattering would be the most efficient method to use. However, for those materials that can be readily isotope substituted to take advantage of one or more elements with isotopes with appreciable coherent cross sections, the poly-CINS method could be used and the signal from the incoherent scattering would be calculated and added to produce a composite *S*′(*Q*, ω). This plot would be used to identify features with no *Q* dependence and exclude them from the search for *Q*-dependent scattering features. Mixed metal hydrides/deuterides would be an example of where poly-CINS would provide a more complete experimental verification of a given model than *via* the incoherent approximation or incoherent inelastic scattering alone.

Although this type of experimental measurement has not been much used in the past, it seems clear that, with the power of modern computational resources, it has the potential to become an important technique for analysing the dynamics of a wide range of intrinsically polycrystalline solids that have coherent cross sections (and incoherent scattering can be simulated to isolate the coherent scattering features for systems with more mixed coherent–incoherent cross sections). In order that this approach be more accessible, the software used for this work will shortly be available in the next release of the *GULP* code, along with the analysis tools (written in Python) used to efficiently identify and compare dispersion curves and theoretical and experimental data sets.

## Supplementary Material

Supplementary material file. DOI: 10.1107/S0021889813023728/he5613sup1.pdf

## Figures and Tables

**Table 1:** Functional forms and associated parameters of the potential models for aluminium

Potential model	Form of EAM functional	EAM density	Pair potential
Sutton–Chen	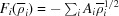 , *A* = 1.000 eV	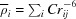 , *C* = 1303.927 Å^6^	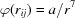 , *A* = 592.41956 eV Å^6^
NP-B	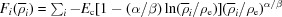 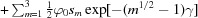 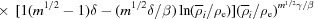 , *E*_c_ = 2.834 eV, α = 4.954, β = 5.203, γ = 5.824, δ = 8.969, ϕ_0_ = 0.2095 eV, *s*_1_ = 6.928, *s*_2_ = 3.861, *s*_3_ = 15.50; ρ_e_ cancels with ρ_e_ in density term	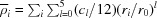 , *c*_0_ = 0.4333, *c*_1_ = −7.305, *c*_3_ = 29.812, *c*_4_ = −54.44, *c*_5_ = −15.50, *r*_0_ = 2.760 Å	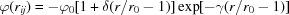 , ϕ_0_ = 0.2095 eV, *r*_0_ = 2.760 Å, δ = 8.969, γ = 5.824
Mei–Davenport	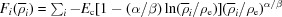 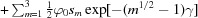 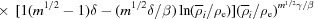 , *E*_c_ = 3.39 eV, α = 4.60, β = 7.10, γ = 7.34759, δ = 7.35, ϕ_0_ = 0.1318 eV, *s*_1_ = 12.0, *s*_2_ = 6.0, *s*_3_ = 24.0; ρ_e_ cancels with ρ_e_ in density term	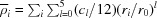 , *c*_0_ = 0.64085, *c*_1_ = −6.83764, *c*_3_ = 26.75616, *c*_4_ = −47.16495, *c*_5_ = −8.60834, *r*_0_ = 2.8638 Å	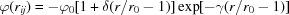 , ϕ_0_ = 0.1318 eV, *r*_0_ = 2.8638 Å, δ = 7.35, γ = 5.58441; MDF taper r(cut) = 5.382 Å; MDF taper f(cut) = 0.522 Å
Lennard-Jones 12–6 potential	N/A	N/A	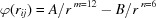 , *A* = 38763.011, *B* = 118.33287

**Table 2:** Experimental and calculated bulk properties of aluminium

	Sutton–Chen	Mei–Davenport	NP-B	Lennard-Jones	Experiment
Lattice constant (Å)	4.050	4.050	4.030	4.050†	4.0495
Binding energy per atom (eV)	−3.33	−3.39	−3.43	−0.52	−3.43
Young’s modulus (GPa)	14.2	32.6	124.0	59.9	70.3
Bulk modulus (Voigt average) (GPa)	75.2	76.87	176.3	68.3	76.9
Shear modulus (Voigt average) (GPa)	9.80	22.9	83.53	35.57	26.1
Poisson’s ratio	0.468	0.429	0.383	0.354	0.345
*C*_11_ (GPa)	82	92	236	98	107
*C*_12_ (GPa)	72	69	146	54	61
*C*_44_ (GPa)	16	37	109	49	29

† For LJ 12–6 this is a fixed value and has not been derived from an optimization.

**Table 3:** Functional forms and associated parameters of the potential models for aluminium, fitted from poly-CINS data

Potential model	Fitted EAM functional parameters	Fitted EAM density parameters	Pair potential parameters
Mei–Davenport	*E*_c_ = 3.335 eV, α = 4.57, β = 7.047, γ = 11.326, δ = 7.35, ϕ_0_ = 0.1318 eV, *s*_1_ = 12.0, *s*_2_ = 6.0, *s*_3_ = 24.0	*c*_0_ = 0.64085, *c*_1_ = −6.83764, *c*_3_ = 26.75616, *c*_4_ = −47.16495, *c*_5_ = −8.60834, *r*_0_ = 2.8638 Å	ϕ_0_ = 0.1330 eV, *r*_0_ = 2.8638 Å, δ = 7.3866, γ = 5.58441
Lennard-Jones (4 Å cutoff)	N/A	N/A	*A* = 28273.896, *B* = 46.590074
Lennard-Jones (12 Å cutoff)	N/A	N/A	*A *= 44389.768, *B* = 135.77357

**Table 4:** Comparison of bulk properties from models fitted to poly-CINS data in this work

	Original Mei–Davenport	Fitted Mei–Davenport	Original LJ 12–6	Fitted LJ 12–6	Experiment
Lattice constant (Å)	4.050	4.050 (4.170)	4.050†	4.050†	4.0495
Binding energy per atom (eV)	−3.326	−3.44 (−3.48)	−0.52	−0.88	−3.43
Young’s modulus (GPa)	14.2	37.1 (37.1)	59.9	44.8	70.3
Bulk modulus (Voigt average) (GPa)	75.2	80.0 (81.9)	68.3	66.5	76.9
Shear modulus (Voigt average) (GPa)	9.80	31.9 (26.8)	35.57	32.85	26.1
Poisson’s ratio	0.468	0.423 (0.425)	0.354	0.388	0.345
*C*_11_ (GPa)	82	97 (99)	98	88	107
*C*_12_ (GPa)	72	71 (73)	54	56	61
*C*_44_ (GPa)	16	44 (36)	49	44	29

† For LJ 12–6 this is a fixed value and has not been derived from an optimization.

## Footnotes

1 Supplementary information is available from the IUCr electronic archives (Reference: HE5613). Services for accessing this material are described at the back of the journal.
