# Supplementary material for: The interpretation of polycrystalline coherent inelastic neutron scattering from aluminium
Source: J Appl Crystallogr. 2013 Oct 26;46(Pt 6):1755–70. doi: 10.1107/S0021889813023728 (PMC3831299; doi:10.1107/S0021889813023728)
Supplement: Supplementary file 1 [file j-46-01755-sup1.pdf]

Supplementary Information: The Interpretation of Polycrystalline Coherent Inelastic Neutron Scattering from Aluminium.

This document provides clarification and additional information in support of the main paper.

Projection of dispersion curves

As stated in the text, and summarised here, the basis for the identification of scattering features is that of the application of the coherence condition (see sections 2.4 and 4.1 of the main text) to polycrystalline samples. Hence polycrystalline S(Q,ω) data is, in effect, a superposition of a multitude of ‘dispersion curves’ over all possible orientations of the crystallites in the sample. The difficulties in identifying which dispersive scattering features correspond to any given powder averaged feature can be overcome by considering the single crystal reciprocal space dispersion surface, as presented in Fig S1, for modes 1, 2 and 3 of the aluminium system.

Figure S2 shows the cut through the compound dispersion surface (all three modes in fig. S1) for the vector joining (000) and (420) reciprocal lattice points, that naturally follows on from fig. 4 in the main text. Fig.S3 shows the approach applied to all of the tau vectors used in this work, to aid in the visual inspection and identification of these features; the crystallographic directions covering the first eleven tau vectors corresponding to the first eleven Bragg peaks allowed in an fcc structure are projected in Q out to the corresponding range used for the (Q, ω) space sampled. This process then leads naturally on to a superposition of the projected curves presented in Fig.S3 in order to generate an overlay plot, as given in Fig.S4. Whilst the application of Fig. S4 as a means of identifying key features is no longer a current part of the analysis – software scripts have been developed to quickly identify the nearest dispersion curve to a given feature via a cut through the data - this process was initially developed to help identify such dispersion curves by visual inspection.

Fig. S5 then completes this clarification exercise – in this figure, the experimental and theoretical features are reduced to points on a projected magnitude of Q scale. In this figure, the experimental features are compared to the fitted dispersion curves for the

eleven tau vectors used in the paper, calculated using the Lennard-Jones 12-6 (4 A^{-1} cutoff).

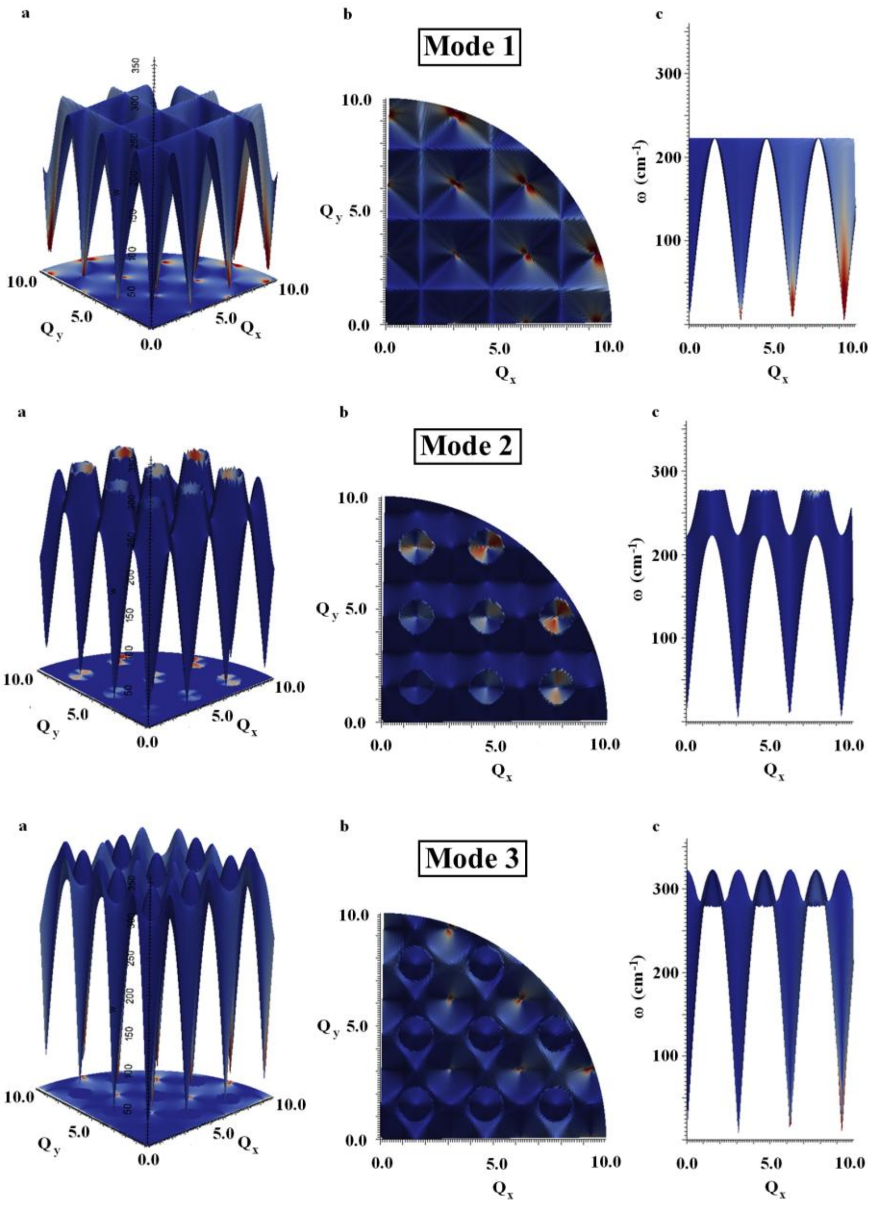

Figure S1: Views of the dispersion surface in the Q_x, Q_y plane of the reciprocal lattice of aluminium, using the original Lennard-Jones potential (before fitting has been applied), with scattering intensity superimposed upon the frequency surface as a colour map. a) shows the full dispersion surface data set as an isometric projection. b) shows the view looking down on the plane. c) shows a cross section through the plane from the Q_x axis perspective. Views are provided for mode 1, mode 2 and mode 3 respectively (Mode numbers assigned low to high frequency).

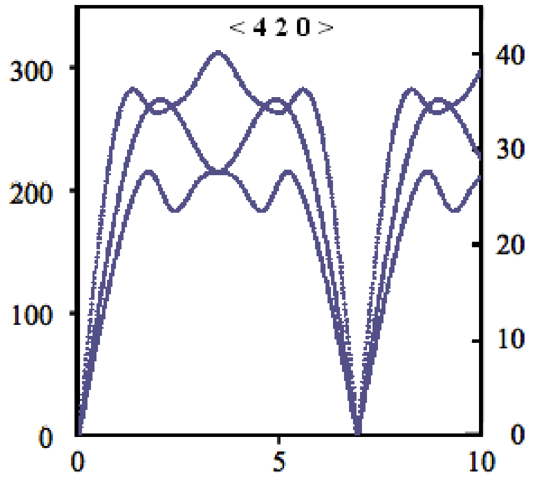

Figure S2: Cut through the dispersion surfaces given in fig. S1, along the vector joining (0 0 0) to (4 2 0), as illustrated in Fig. 4c in the main text.

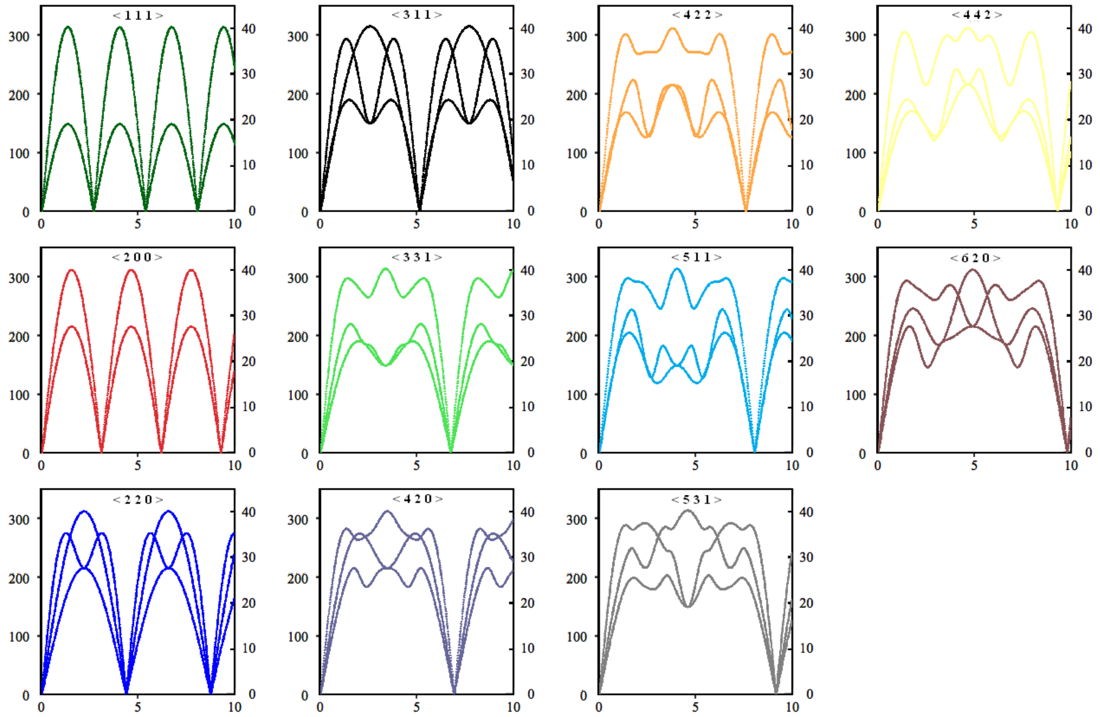

Figure S3. Projections of the calculated dispersion curves of Aluminium (due to LJ12-6 model) into momentum transfer Q for the first eleven allowed reciprocal lattice directions.

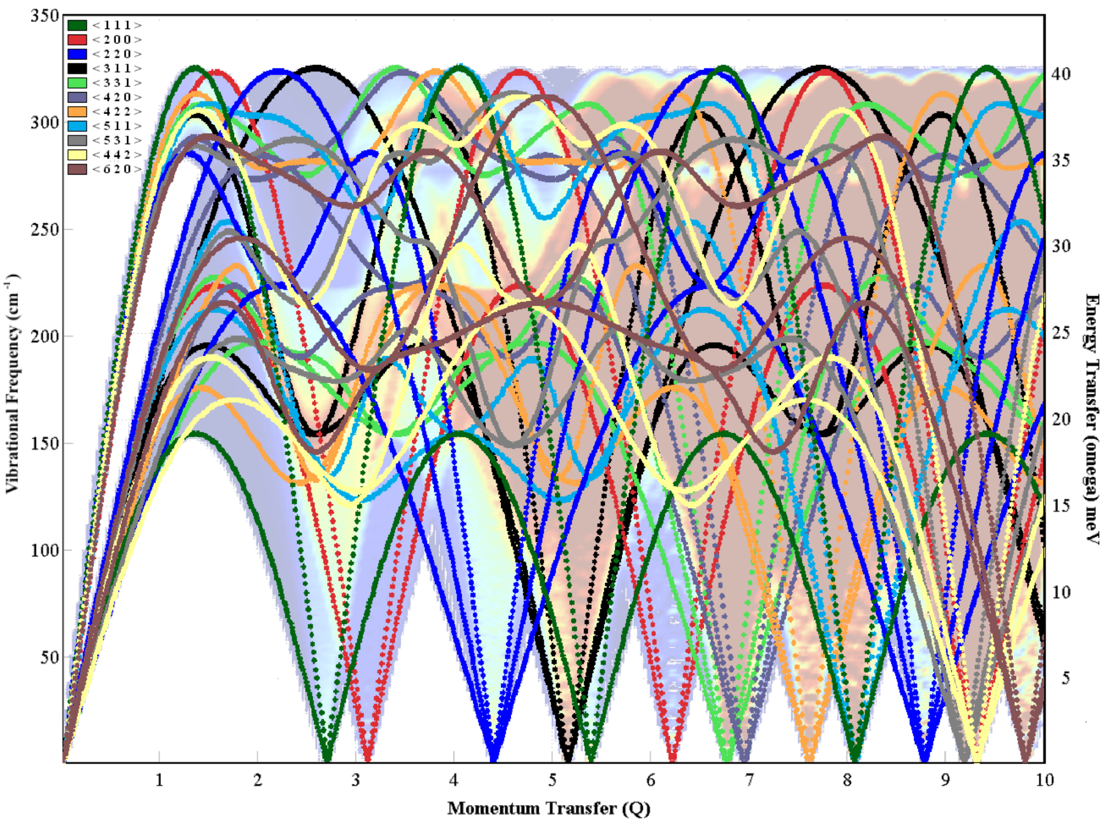

Figure S4: Projections of the calculated dispersion curves of Aluminium into momentum transfer Q, overlaid onto calculated S'$_{coh}$(Q,ω), illustrating the origin of major features in the poly-CINS scattering.

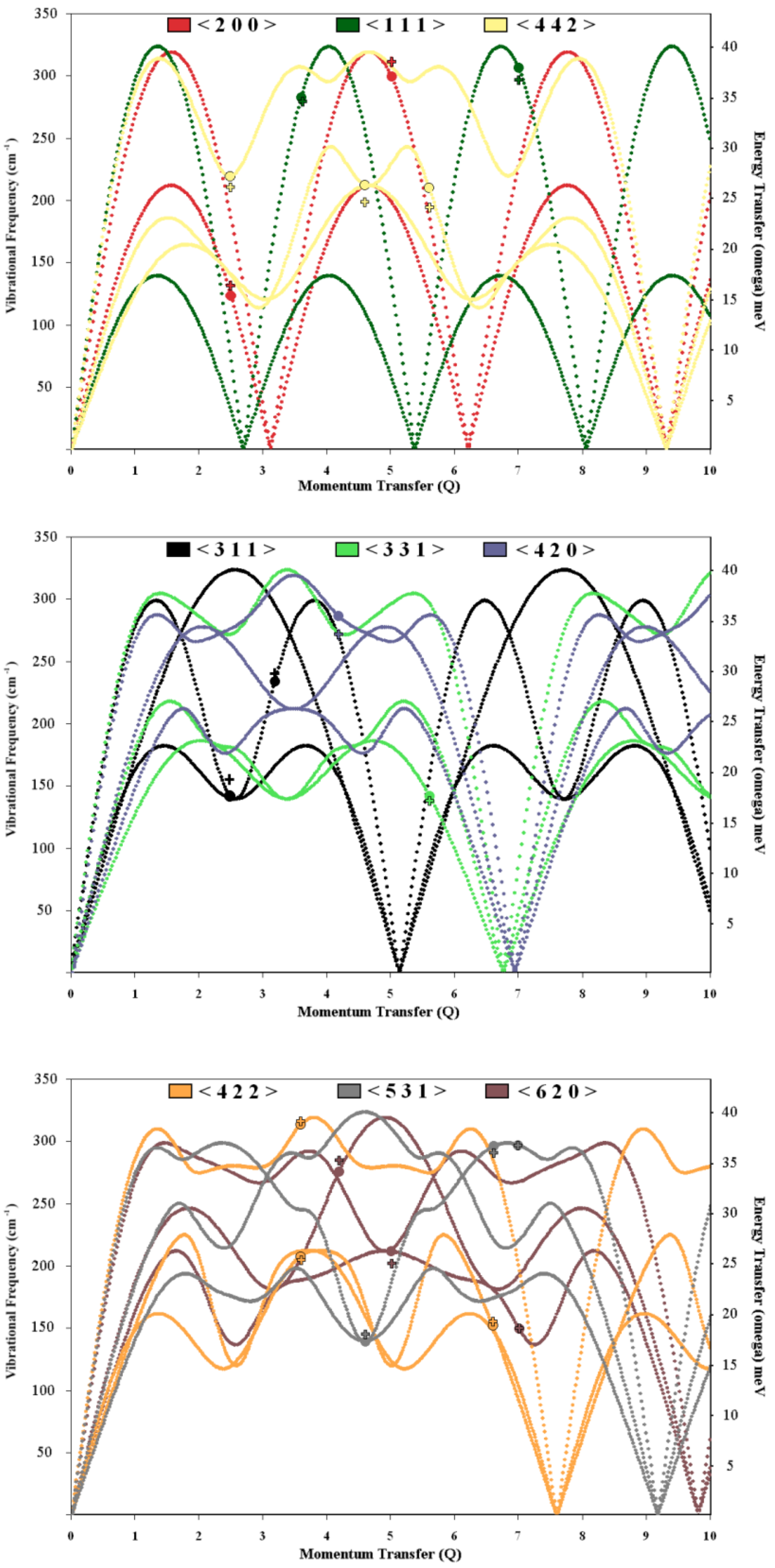

Figure S5: Cuts through the dispersion surfaces featured in S1 and S3, showing the LJ-12-6 model fitted to experimental data (as in Fig.8 in main text). Circles denote the theoretical points (red in Fig.8) and the crosses the experimental data points (black in fig.8).
